# Fellowship Away from Home: A Journey toward Advancing the Current Practice of Reconstructive Surgery in Ethiopia

**DOI:** 10.1055/a-2039-3653

**Published:** 2023-03-31

**Authors:** Abeje Brhanu Menjeta

**Affiliations:** 1 Department of Plastic and Reconstructive Surgery, St. Paul's Hospital Millennium Medical College, Addis Ababa, Ethiopia

**Figure:**
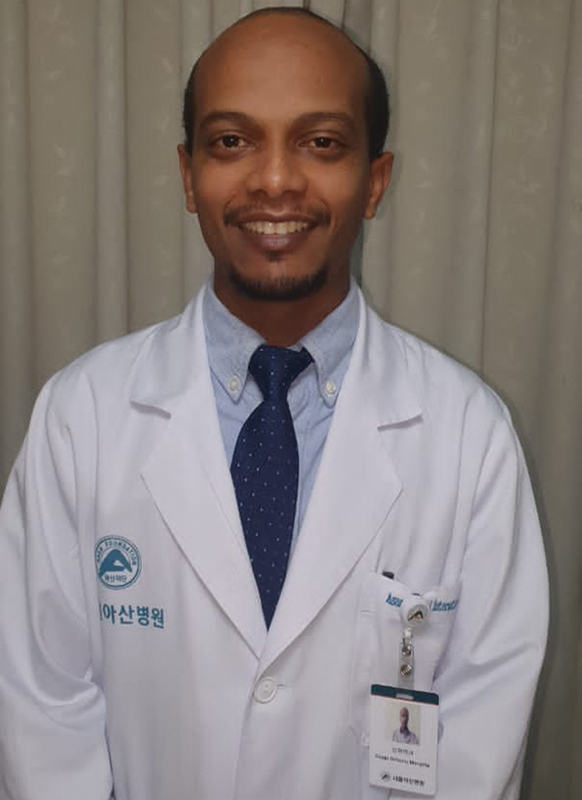
Abeje Brhanu Menjeta, MD, FCS(ECSA)

It has been declared by many and mentioned in number of ways that our achievements and impacts in life are significantly dependent on the type of journey that we choose to take and how further and better we travel on that chosen journey. Likewise, there is an Ethiopian quote which says “When you go further on a life journey, you will encounter with thousands of opportunities.” In the same spirit as the above quote, a plastic and reconstructive surgery fellowship at Asan Medical Center allowed me to travel further on the fascinating journey of reconstructive surgery. A journey which is very much unique and unattempted by many plastic surgeons living in the part of the world where I come from, Africa. On this expedition I happen to encounter with several opportunities to be exposed to a variety of knowledge, skills, and attitudes regarding reconstructive surgery. An opportunity to challenge myself into acquiring of new sets of skills and practices in plastic and reconstructive surgery in general and reconstructive microsurgery in particular. An opportunity to meet and create network with several prominent figures in the field of reconstructive surgery. An opportunity to get exposed to new language, culture, and weather conditions. All the opportunities that I encountered by going further on the highway of reconstructive surgery is made possible due to this fellowship. This opportunity and the associated exposure that I run into due to the fellowship, have already made me to be a much better plastic and reconstructive surgeon with several added values in terms of knowledge, skills, and attitude. I should also admit that the fellowship has also created the opportunity for me to broaden my view toward new cultures and the untaken roads.

A persistent and strong personal desire to be one of change catalysts for the advancement of reconstructive surgery in Ethiopia is the main motivational factor which serve as a drive for embarking on this journey of fellowship. The current practice of plastic and reconstructive surgery in Ethiopia is characterized by usage of old-fashioned methods of reconstruction, where skin graft, local flap, and pedicled regional and distant flaps are used as the main reconstruction armamentariums. Reconstructive microsurgery is one of the missed tools in our current practice of reconstructive surgery.

Even if I am among the very few from my region who happens to be lucky to take the journey of fellowship in reconstructive microsurgery, I am very sure that the impact of this astonishing journey will not be confined to an individual level. The story of my journey will be used as a source of hope and inspiration for many young plastic surgeons who wish to go further in the field of reconstructive surgery. In addition, I also like to assure that all the skills and knowledge which I happen to gather in this fellowship will be shared enthusiastically to all my colleagues and students for initiating transformation in the field of reconstructive surgery in my country and region.

I would like to thank the honorable professor K.S. Koh for his service as a dedicated and exemplary reconstructive surgeon and for being one of the reasons behind my fellowship. I also like to thank Professor J.P. Hong for his relentless mentoring and support in this journey of fellowship in plastic and reconstructive surgery.

